# Direct derivation of maize plant and crop height from low-cost time-of-flight camera measurements

**DOI:** 10.1186/s13007-016-0150-6

**Published:** 2016-11-28

**Authors:** Martin Hämmerle, Bernhard Höfle

**Affiliations:** 1 GIScience Research Group, Institute of Geography, Heidelberg University, Im Neuenheimer Feld 368, 69120 Heidelberg, Germany; 2 Heidelberg Center for the Environment (HCE), Heidelberg University, 69120 Heidelberg, Germany

**Keywords:** Precision agriculture, Site-specific crop management, Continuous raster crop height model, Individual plant height, 3D geodata, Low-cost time-of-flight camera

## Abstract

**Background:**

In agriculture, information about the spatial distribution of crop height is valuable for applications such as biomass and yield estimation, or increasing field work efficiency in terms of fertilizing, applying pesticides, irrigation, etc. Established methods for capturing crop height often comprise restrictions in terms of cost and time efficiency, flexibility, and temporal and spatial resolution of measurements. Furthermore, crop height is mostly derived from a measurement of the bare terrain prior to plant growth and measurements of the crop surface when plants are growing, resulting in the need of multiple field campaigns. In our study, we examine a method to derive crop heights directly from data of a plot of full grown maize plants captured in a single field campaign. We assess continuous raster crop height models (CHMs) and individual plant heights derived from data collected with the low-cost 3D camera Microsoft^®^ Kinect^®^ for Xbox One™ based on a comprehensive comparison to terrestrial laser scanning (TLS) reference data.

**Results:**

We examine single measurements captured with the 3D camera and a combination of the single measurements, i.e. a combination of multiple perspectives. The quality of both CHMs, and individual plant heights is improved by combining the measurements. R^2^ of CHMs derived from single measurements range from 0.48 to 0.88, combining all measurements leads to an R^2^ of 0.89. In case of individual plant heights, an R^2^ of 0.98 is achieved for the combined measures (with R^2^ = 0.44 for the single measurements). The crop heights derived from the 3D camera measurements comprise an average underestimation of 0.06 m compared to TLS reference values.

**Conclusion:**

We recommend the combination of multiple low-cost 3D camera measurements, removal of measurement artefacts, and the inclusion of correction functions to improve the quality of crop height measurements. Operating low-cost 3D cameras under field conditions on agricultural machines or on autonomous platforms can offer time and cost efficient tools for capturing the spatial distribution of crop heights directly in the field and subsequently to advance agricultural efficiency and productivity. More general, all processes which include the 3D geometry of natural objects can profit from low-cost methods producing 3D geodata.

**Electronic supplementary material:**

The online version of this article (doi:10.1186/s13007-016-0150-6) contains supplementary material, which is available to authorized users.

## Background

Information about crop height and its spatial distribution is of high value for agriculture. By including this information into the management and field work processes, agricultural productivity and efficiency can be improved [1], which in turn can be a means of improving global food supply and of tackling challenges related to climatic changes [2, 3].

Examples for the usage of crop height models (CHMs) are site-specific crop management [4, 5], plant nitrogen estimates [6], and yield and biomass estimations [7–9]. In addition to CHM raster models continuously covering a whole crop stand, the height of an individual plant at is of high value for agricultural research. Freeman et al. [10], for example, present a high correlation between maize plant height and biomass. Similar, models for corn yield estimation are improved by including plant height [11, 12], and Muharam et al. [13] state significant correlations between plant height and nitrogen nutrition status.

Approaches for a non-invasive collection of 3D geodata as a basis for deriving crop height models vary widely [14]. High-end airborne laser scanning (ALS) is used to capture the crop height of maize fields in the study presented by Li et al. [15], with high correlations stated between the ALS data and manual field measurements. Friedli et al. [16] apply terrestrial laser scanning (TLS) to monitor crop growth, and Crommelinck and Höfle [17] examine the requirements in terms of TLS sensor resolution for deriving CHMs, aiming at low-cost devices for permanent crop monitoring. Following a low-cost photogrammetric approach to generate 3D geodata, Li et al. [8] and Bareth et al. [18] present crop surface models generated on the basis of image collections captured from unmanned aerial vehicle platforms. Marx et al. [19] describe subjective crop height data collection using smartphone devices by non-experts and successfully derive seamless crop height models of high quality when compared to TLS reference data. Another approach is suggested in [20, 21] where the crop height is directly derived via the distance between a LiDAR device and the crop surface.

The methods for gathering 3D geodata applied in the mentioned studies have their particular advantages and restrictions. Laser scanners are active systems, not depending on specific lighting conditions. Furthermore, laser beams can penetrate vegetation so that measurements of the terrain are possible in vegetated areas. However, static terrestrial laser scanning is prone to occlusion of the terrain by vegetation and can include unfavorable scanning geometries [17]. Airborne laser scanning offers an advantageous perspective close to nadir which minimizes occlusion of the terrain by plants, but the method comprises restrictions in terms of temporal and spatial resolution. Regarding photogrammetric approaches, data acquisition and derivation is straight forward, but the sensors are passive and subsequently sensitive to different lighting conditions. Furthermore, crops can occlude the terrain in photogrammetrically analyzed images, which leads to restrictions in terms of seamless crop height derivation [22].

Additionally, most of the mentioned studies comprise at least two field campaigns, one for capturing data of the terrain without vegetation, and subsequent campaigns for capturing the crop surface. Contrary, Li et al. [15] and Luo et al. [23] profit from ALS measurements reaching the terrain through gaps in the crop canopy and successfully achieve a direct CHM derivation from only one measurement campaign. Similarly, Grenzdörffer [22] tests a direct CHM derivation from low-cost photogrammetry point clouds, but conclude that this approach is less reliable compared to the usage of a digital terrain model (DTM) captured before plant growth due to the terrain being highly occluded by the crop canopy in the used images.

The motivation for our study draws from the idea of directly deriving crop height models and individual plant heights without the need of a prior DTM, using an active low-cost sensor scanning from a nadir perspective and, subsequently, minimizing terrain occlusion by plants. The device used in this study for capturing 3D data of crops is the time-of-flight 3D camera Microsoft^®^ Kinect^®^ for Xbox One™ (i.e. the second Kinect^®^ generation, in our study abbreviated with ‘K2’). Similar to Marinello et al. [24], who apply the first Kinect generation for dynamic soil surface characterization under field conditions, a setup of K2 devices mounted in nadir perspective on autonomous mobile platforms or in arrays along booms of agricultural machines can be imagined, offering a time and cost efficient tool for capturing the distribution of crop heights directly in the field.

The aim of this study is to assess (1) raster crop height models and (2) individual plant heights directly derived from K2 data without prior measurements of the bare soil. The CHMs and plant heights are calculated from single and combined K2 measurements to examine the improvement of derivatives via the combination of multiple K2 perspectives. A TLS dataset provides the reference for comparing the CHMs on a raster cell level and the plant heights on a point cloud level. We address advantages and limitations of using the K2 especially for capturing vegetation objects such as agricultural crops.

## Methods

**Fig. 1:**
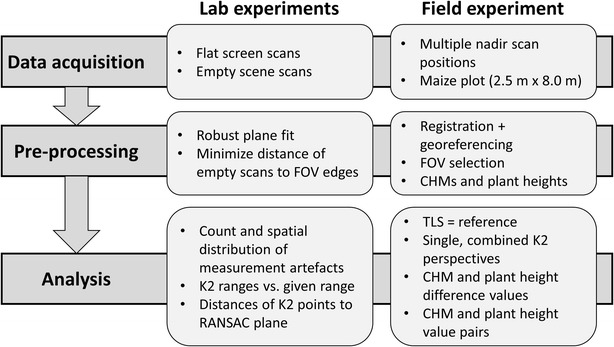
Workflow summing up the major steps for assessing crop height models and individual plant heights derived from K2 data

Our study comprises lab experiments and the analysis of point clouds captured in the field (Fig. 1). In the lab experiments, we examine the performance of the used K2 sensor in terms of precision, accuracy and occurring measurement artefacts on the basis of scans of a flat screen and of empty scenes in different lighting conditions. The field data consists of K2 and TLS point clouds of a maize field. From the point clouds, crop height models and individual plant heights are derived, and the K2 data are comprehensively compared to the TLS reference.

### The Kinect^®^ for Xbox One™ sensor

The K2 measures the distances between sensor and objects within a field of view (FOV) of 70° × 60° by actively emitting a near infrared signal (850 nm) and measuring the time shift between signal emission and backscattered signal detection for each of the 512 × 424 sensor pixels. With the resulting point cloud consisting of 217,088 XYZ coordinates derived from a depth image, distances from 0.5 to 4.5 m can be covered [25–27]. Calculated from FOV angles and the number of sensor pixels, both the horizontal and vertical theoretical resolution (spacing of range measurements) range from 0.0014 m at 0.5 m scanning range to 0.0109 m at 4.0 m scanning range. For our study, the device was operated with the software toolkit KinectPV2 [28].

### Performance of K2 sensor

To assess the performance of the K2 sensor, experiments are performed under controlled conditions (Fig. 1). We examine precision (repeatability), accuracy (conformity of measurements to true value), and measurement artefacts in the form of 3D coordinates recorded in a completely empty scene as produced by the sensor.

To test the device for measurement artefacts, an empty scene is measured 100 times under four different lighting conditions (night, in diffuse light i.e. shadow, direct sunlight with the sensor facing away from the sun, direct sunlight with the sensor facing directly into the sun). The distribution of measurement artefacts within the sensor’s FOV is assessed by calculating the distance of each recorded XYZ coordinate to a mesh of the FOV edges (Additional file 1) after minimizing the distance between the K2 point clouds and the FOV edges via the iterative closest point algorithm [29, 30]. The mean number of measurement artefacts is derived, and their spatial distribution is assessed by calculating the median, maximum, and standard deviation of the distances between the points and the FOV edges.

Precision and accuracy of the device used in this study are examined on the basis of K2 measurements of the center of a planar screen [26, 31]. K2 measurements of the screen are taken from 0.5 to 4.0 m distance in 0.5 m steps, covering the minimum measurement range given by the manufacturer [27] and the maximum scanning range applied in our field study. The point cloud captured from 0.5 m distance contains a data gap of approximately 50% in the center area of the point cloud so that, additionally, one dataset was captured from 0.80 m distance, which was found to be the minimum distance to provide a seamless point cloud of the measured area. To exclude pincushion distortion effects at the outer edges of K2 measurements [26], only the inner third of the K2 field of view is considered for the experiment. The precision of the K2 device is expressed as standard deviation (SD) of residual distances to a plane fitted into the point cloud via a robust random sample consensus (RANSAC) algorithm. The accuracy is examined via the root mean square of differences between given range and the mean of actually measured range values (RMSE) [26].

### Direct derivation of crop height models

**Fig. 2:**
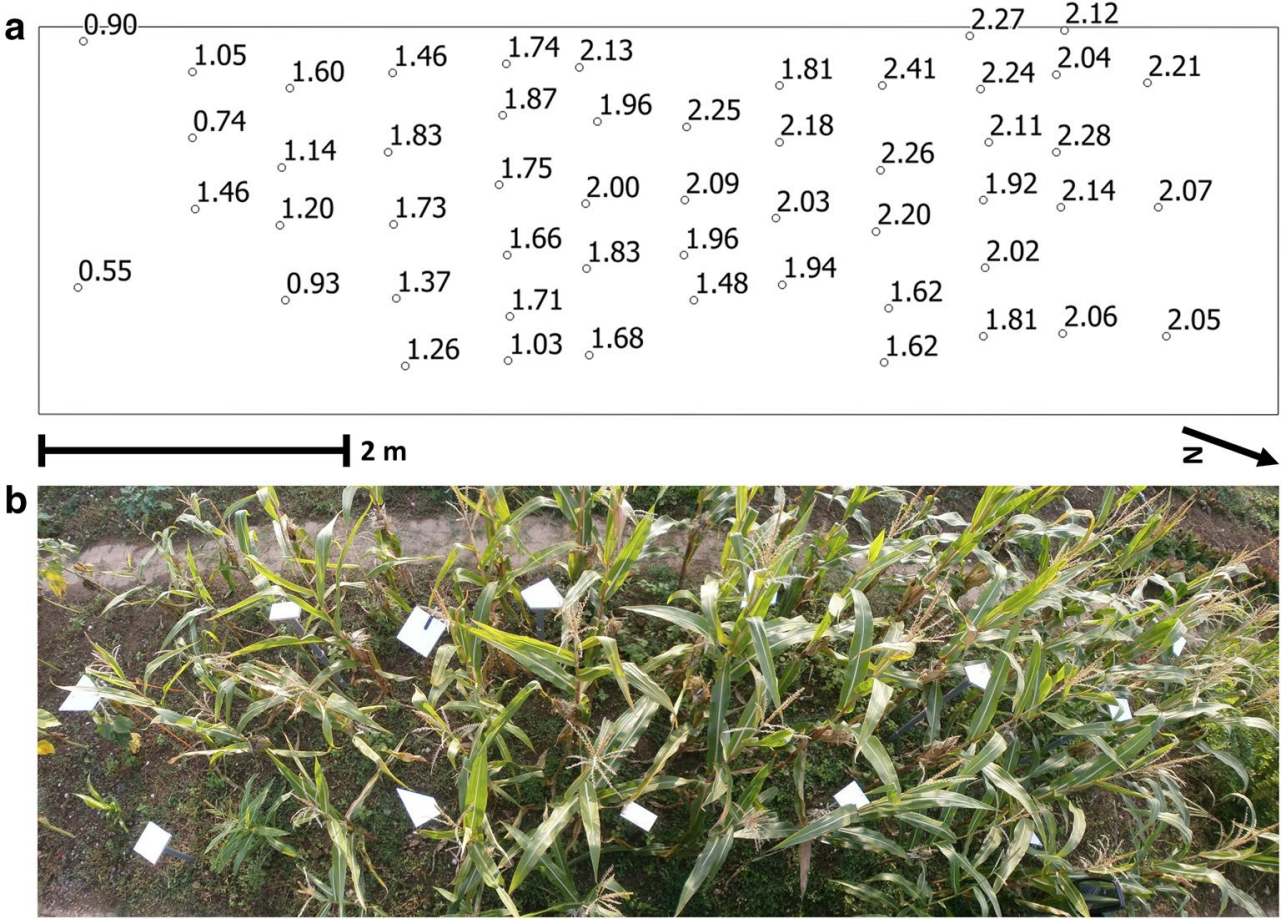
Distribution of the maize plants and 3D registration markers within the study area. **a** Individual plant positions labeled with plant heights extracted from TLS data. **b** Panoramic bird’s eye view onto the field and the white 3D registration markers

The study area for tests of direct crop height derivation consists of a planar maize plot with an extent of 2.50 m × 8.00 m (Fig. 2). The field campaign took place on September 21st, 2015, in totally calm weather and twilight between 16:00 and 21:00 UTC+1 to avoid the movement of plant parts and direct sunlight interfering with the K2 signal. Within the field, 52 plants ripe for harvesting are distributed regularly with a mean longitudinal distance of 0.58 m and a mean transverse distance of 0.46 m. Due to shadowing by a tree standing south of the field, our study comprises plants with various heights, ranging from minimum 0.55 m in the southern part to maximum 2.41 m in the northern part. To transfer the K2 and TLS measurements into the same coordinate system (i.e. to register the datasets), stable 3D registration markers are placed in different heights within the maize field.

**Fig. 3:**
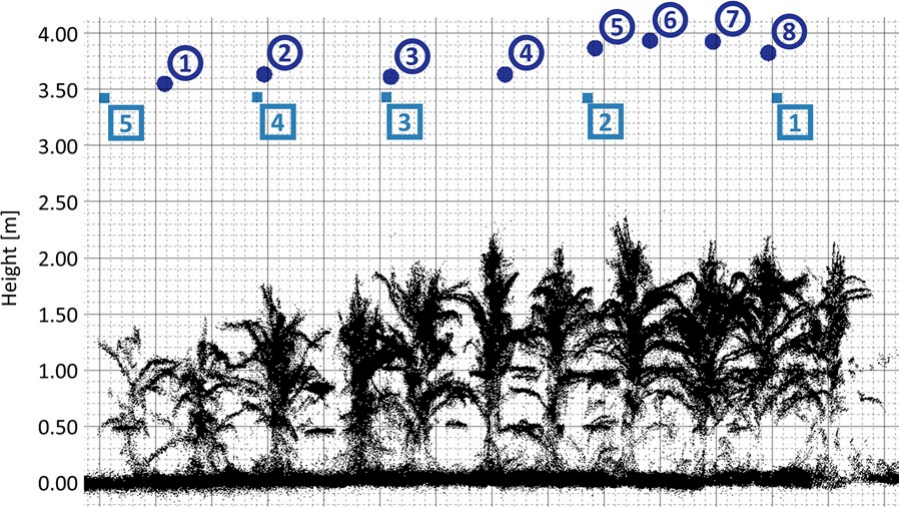
Side view on the combined K2 point clouds covering the maize field. *Blue circles* K2 scan positions, *blue rectangles* TLS scan positions

According to the idea of having a K2 device mounted on agricultural machines looking vertically downwards into a crop stand, the K2 is fixed on a pole and positioned parallel to the long edge of the field in a nadir perspective (Additional file 2). To cover the whole field with high overlap, K2 point clouds are captured from 8 positions from in average 3.75 m height above ground (Fig. 3). Position 1 is located in the southern part of the plot containing the low plants, position 8 is located in the northern part with the highest plants.

To capture also small parts of the maize plants, the applied terrestrial laser scanner Riegl VZ-400 collected the reference dataset at a high horizontal and vertical angular resolution of 0.029°, corresponding to a point spacing of 2.5 mm at 5 m scanning range. The TLS device offers a range measurement precision of 3 mm and an accuracy of 5 mm at 100 m scanning range [32], and it was mounted approximately 3.50 m above ground. To account for the device’s field of view restriction of 50° relative to nadir, the scanner was mounted on a tilted platform (Additional file 2). To cover the ground completely, the field was scanned from 5 TLS scan positions (Fig. 3).

To prepare both the K2 and the TLS data for the analyses, several pre-processing steps are applied (Fig. 1). First, the TLS point clouds are registered and georeferenced. The single TLS scan positions are registered by means of corresponding tie points which were manually defined at distinct corners of the 3D markers. The registration of the single TLS scan positions is achieved via 11–21 tie point pairs, resulting in a standard deviation of 0.20–0.35 cm for the residual 3D distances between the used tie points. The mean cloud-to-cloud distances on selected 3D marker surfaces range from 0.10 to 0.30 cm. Additionally, overlapping areas on stable objects such as the 3D registration marker pipes are visually inspected regarding shifts between the point clouds, and also the visual control indicates a high TLS registration quality.

To level and georeference the TLS data, a 3D transformation is applied with parameters for translation and rotation. These are derived by picking the local coordinates of 9 distinct maize plant positions in the registered TLS point cloud and by subsequently linking the local coordinates to their respective global coordinates surveyed with a high-end RTK GNSS Leica Viva GS10/GS15. A standard deviation of 3D distance residuals of 3.00 cm is achieved, being a valid result especially regarding the sole aim of the georeferencing step, i.e. the leveling of the TLS data.

The workflow of the second pre-processing step, i.e. the co-registration of the K2 point clouds onto the TLS reference data, corresponds to the process used for TLS registration. For the co-registration, 5–14 tie point pairs are used. The standard deviation of residual 3D distances ranges from 0.50 to 1.60 cm. The mean cloud-to-cloud distances between 3D markers in the TLS and K2 data are between 0.20 and 3.00 cm. The achieved registration and co-registration accuracy has to be kept in mind when interpreting the comparison between TLS and K2-based CHMs.

To exclude data of plants not being completely within the FOV and subsequently not being relevant for CHM or plant height derivations, each K2 point cloud is clipped to the extent of the FOV at the distance between sensor and the highest plant within the measured scene (Additional file 3). Finally, all point clouds are clipped to the area of interest with an extent of 2.5 m × 8.0 m. To exclude measurement artefacts from the point clouds, a statistical outlier filter [33] is applied on both the TLS and the K2 point clouds. The filter removes all points which are spatially isolated in terms of the mean distance to the five closest neighbors being larger than the standard deviation of the distances.

The final number of points after cropping the data to the FOV extent and after applying a statistical outlier removal is given in Table 1. The K2 point clouds are considerably reduced by removing all points on the FOV edges and excluding the points outside the extent of the upper FOV rectangle (Additional file 3).

**Table 1:** Number of points in the original data and the final point clouds achieved after outlier removal

Sensor	Scan position	Number of points			
		Original point cloud	Cropped to FOV	After SOR filter	% of original point cloud
TLS	All combined	17,812,208	Not applied	16,351,675	91.80
Kinect 2	1	217,088	70,430	66,872	30.80
Kinect 2	2	217,088	66,814	61,169	28.18
Kinect 2	3	217,088	64,386	59,697	27.50
Kinect 2	4	217,088	57,741	52,809	24.33
Kinect 2	5	217,088	61,540	57,486	26.48
Kinect 2	6	217,088	73,863	69,121	31.84
Kinect 2	7	217,088	73,653	68,370	31.49
Kinect 2	8	217,088	62,628	59,972	27.63

The pre-processed point clouds are the basis for deriving 11 CHMs: 1 CHM for each of the 8 single scan K2 positions, partly covering the maize plot according to the respective FOV, and 3 CHMs for the combined scan positions 1-3-5-7, 2-4-6-8, and 1-8, extending over the whole maize plot. The TLS reference CHMs are calculated on the basis of all TLS point clouds combined in order to achieve the best possible coverage of ground and plants.

The crop height models of 0.25 m × 0.25 m cell size are derived with the software package OPALS [34] by normalizing a digital surface model (DSM) with a digital terrain model (DTM). The raster cell size was chosen based on plant spacing in order to achieve a seamless CHM and to avoid multiple plant tips within the same raster cell. The DSMs are derived by assigning maximum elevation of all points within a raster cell to the respective cell value [17]. The DTM values correspond to the lowest point within the respective cell. The outermost cells of each CHM are removed to exclude cells covered only partly by the point clouds. The final CHMs consist of 42–246 raster cells.

The accuracy of the CHMs derived directly from K2 point clouds is assessed via a set of measures as recommended by Höhle and Höhle [35]. The measures are based on the difference values between the K2 CHM and the TLS reference CHM. Subsequently, difference raster cells with positive values occur in case the K2 CHM is higher than the TLS reference CHM, and negative values occur if the K2 CHM is lower than the TLS reference CHM. First, the distribution of errors is visually assessed in terms of normality with histograms and quantile–quantile (Q–Q) plots. Subsequently, the root mean squared error (RMSE), mean and standard deviation are calculated for the CHM differences. Mean and SD are also derived from the differences excluding all blunders, i.e. CHM differences with absolute values over three times the RMSE. Additional robust accuracy measures are derived and compared in order to account for blunders: The quantiles for 95, 68.3 and 50% (i.e., the median), and the normalized median absolute deviation (NMAD), calculated with

$$NMAD = 1.4826 \times median\left( {\left| {\Delta h_{i} - median_{{\Delta h}} } \right|} \right)$$

To assess the CHM accuracies also on a cell-level, scatter plots are derived for corresponding raster cell values for the TLS and K2 CHMs. A linear function is fitted into the data pairs and the coefficient of determination R^2^ is derived as a basis for the assessment.

### Direct derivation of plant heights

In addition to the CHM analyses, individual plant heights are derived directly from the point clouds. The plants are extracted according to two scenarios: (1) the K2 point clouds are not georeferenced and measurements are available only for one specific date, and (2) the K2 point clouds are georeferenced and the plant positions are extracted from measurements at an early plant development stage (e.g., [36]).

To derive the plant height for scenario 1 (i.e., the K2 point clouds are not georeferenced), points representing the local maximum height of the canopy surface are selected. For each local maximum point, the lowest point within a search radius of 0.125 m is extracted. The plant height is subsequently calculated by subtracting the local minimum height from the local maximum height. All difference values below 0.500 m are excluded based on the a priori knowledge that all plants in the maize plot are larger. The extracted K2 plant heights are compared to the nearest local maximum point in the TLS cloud within a radius of 0.125 m. In case of scenario 2 (i.e., the K2 point clouds are georeferenced and the plant positions are known), the local maxima and minima and subsequently the plant heights are extracted from the K2 and TLS point clouds from within a radius of 0.125 m around the known plant position. The plant heights are compared based on the coefficient of determination of linear models fitted into the data pairs, and additionally on the median, standard deviation, and RMSE of plant height difference values.

## Results

### Performance of K2 sensor

The experiment focusing on measurement artefacts via scans of empty scenes results in the statistics given in Table 2. The lighting conditions have a noticeable effect with approximately four times the number of measurement artefacts in daylight when compared to the measurements at night. Regarding the spatial distribution of measurement artefacts, the median values of the distances between measurement artefacts and the FOV edges indicate a concentration of measurement artefacts on the FOV edges. However, large distance values are contained in all measurements, and also the standard deviation is relatively large in all cases which means that also some measurement artefacts occur within the volume delimited by the FOV edges. Especially in case of the measurements taken with the sensor directly facing into the sun, a column of measurement artefacts occurs in direction of the sun. Based on visual inspection of the single K2 point clouds, it can additionally be stated qualitatively that the distribution of measurement artefacts is randomly changing with every measurement.

**Table 2:** Results of the measurement artefact experiments (100 measurements per lighting condition)

Lighting conditions of scanned empty scene	Average count of measurement artefacts (SD)	Distances between measurement artefacts and FOV edges (m)		
		Median	Max.	SD
Night	50 (7.4)	0.009	1.46	0.17
Diffuse sunlight	195 (14.4)	0.013	1.54	0.11
Direct sunlight facing away from sun	202 (10.1)	0.007	1.50	0.11
Direct sunlight facing into sun	218 (13.4)	0.014	1.51	0.20

The lab experiments for determining precision and accuracy of range measurements result in precision values from 0.001 m at 0.80 m distance to 0.003 m at 4.0 m distance. The RMSE values representing accuracy range from 0.005 m (0.80 m distance) to 0.024 m (4.0 m distance).

### Direct derivation of crop height models

**Fig. 4:**
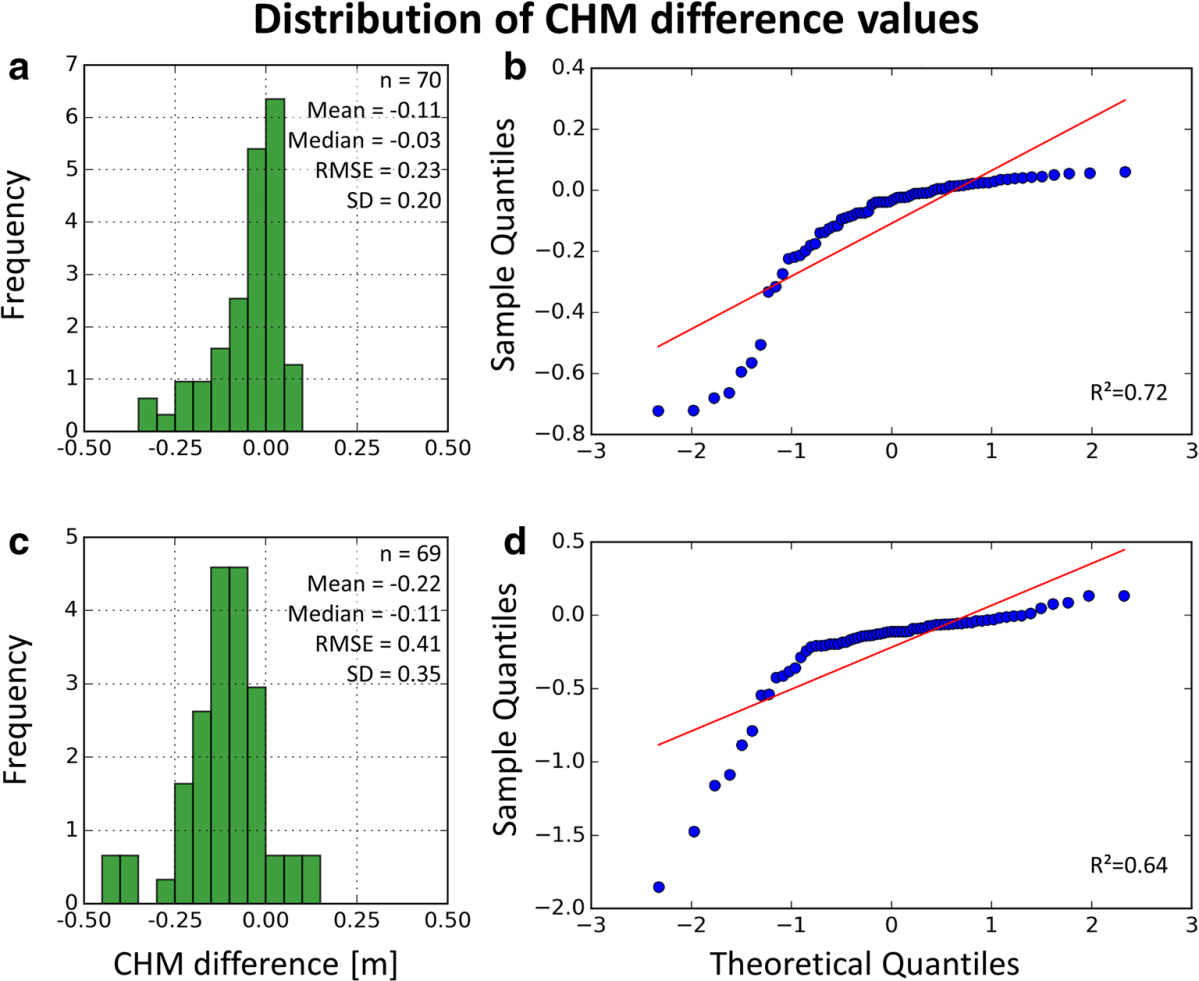
Distribution of CHM differences for the CHMs with minimum (K2 scan position 1) and maximum (K2 scan position 7) median difference. **a** Histogram for K2 scan position 1. **b** Q–Q plot for scan position 1. **c** Histogram for scan position 7. **d** Q–Q plot for scan position 7

**Fig. 5:**
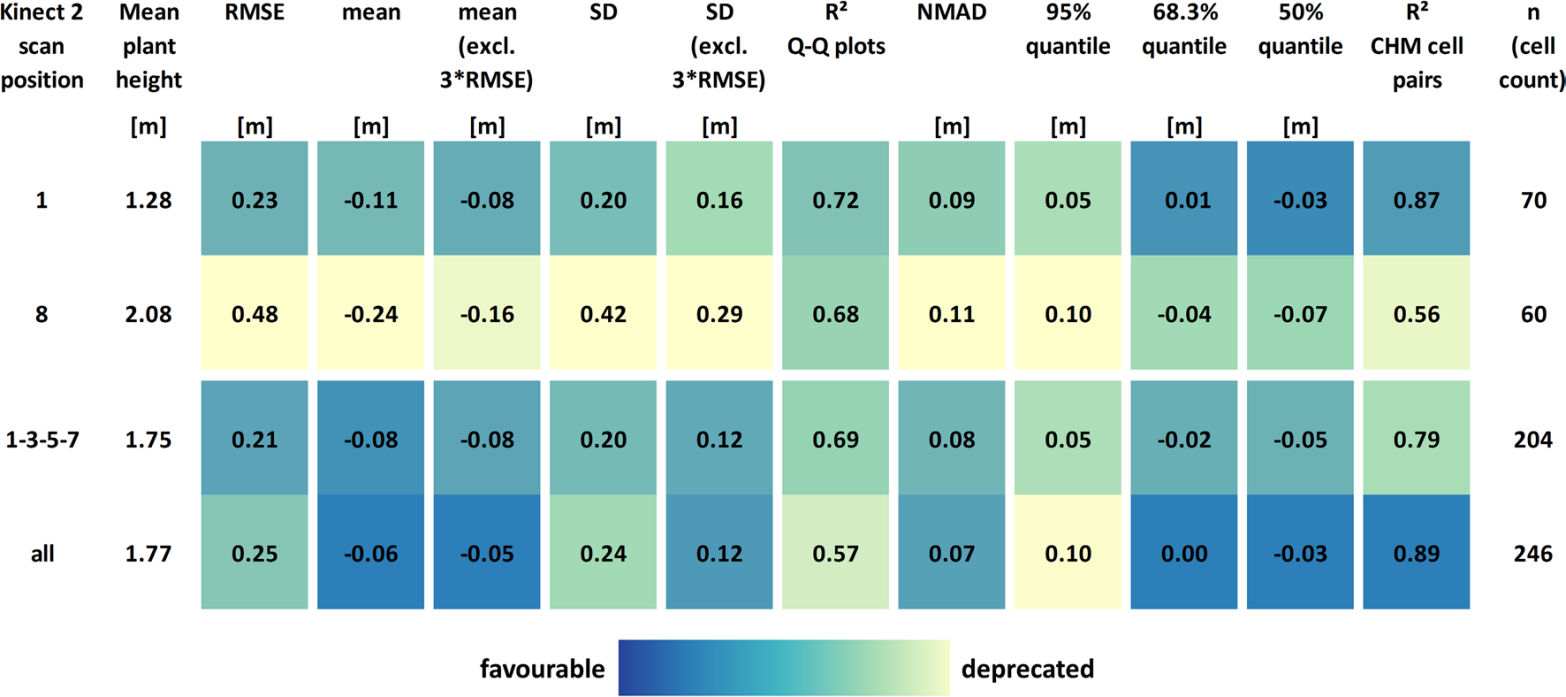
Selection of CHM accuracy measures derived from the difference values CHM_K2_ − CHM_TLS_. *Rows* K2 scan position, *columns* accuracy measure

Figure 4 shows two exemplary histograms and Q–Q plots selected according to the smallest and largest median of CHM differences. All difference values of the 11 examined CHMs are close to a unimodal normal distribution with an acute peak around the mean value, at the same time having heavy tails due to blunders. The Q–Q plots indicate a normal distribution including blunders which lead to a deviation from the linear shape of an ideal Q–Q plot. The distributions show a tendency towards a negative skew, with large CHM differences occurring in the left tail pointing at a crop height underestimation in the CHMs derived from K2 data. This is also reflected in all mean and median values being below zero (Fig. 5), indicating that the CHMs derived from K2 data are generally lower than the TLS reference.

The calculated accuracy measures are summed up for scan positions 1 (low plants), 8 (high plants), 1-3-5-7 combined and all combined in Fig. 5 (for the complete list covering all scan positions see Additional file 4). The RMSE of the single K2 point clouds increases in accord with the increasing number and magnitude of blunders stated above (Fig. 4), whereas the RMSEs of combined point clouds tend to be lower. However, large CHM difference values also occur for the combined point clouds, ranging from −1.65 to 1.17 m in case of the CHMs derived from all point clouds combined.

The mean CHM differences are negative in all cases, also when excluding CHM difference values larger than three times the RMSE. The largest values for mean deviation occur for scan positions in the plot area with higher plants, where more and larger blunders occur in the CHMs. Assuming an average plant height of 2.08 m for the area captured from SP8, a mean CHM underestimation of 11.54% is calculated whereas in best case (all point clouds combined), the mean CHM difference results in an underestimation of 3.39% assuming an average plant height of 1.77 m for the covered area. Also regarding the standard deviation, combining K2 point clouds captured from different scan positions results in lower values compared to almost all of the single point clouds. The highest R^2^ of Q–Q plots is derived for the CHM of scan position 2, indicating the best correspondence of the CHM difference value distribution to a normal distribution, whereas the values derived from the point clouds of other scan positions are lower due to cells containing pronounced CHM underestimations.

To achieve an accuracy assessment more robust against blunders, Höhle and Höhle [35] recommend comparisons based on quantiles and NMAD. Compared to the 68.3% quantile values, the NMAD is larger for all CHMs. Differences are largest for two point clouds captured in the area with highest plants. The 95% quantiles are more than two times larger than the 68.3% quantile in 5 of 11 cases (scan positions 2–6) which can be attributed to the occurrence of pronounced CHM differences [35].

The median CHM differences (i.e., the 50% quantiles) range from −0.11 m (scan position 7) to −0.03 m (scan position 1). The CHM difference values of all datasets except for scan position 7 can be regarded as being within the performance of the sensors in terms of precision and accuracy. The tendency stated above for the K2-based CHMs to underestimate crop height is reflected in all of the median values being negative.

The R^2^ of corresponding CHMs offers an insight into the CHM quality on a cell level. In accordance with the other accuracy measures, combining the single K2 point clouds again results in a strongly improved R^2^. When regarding only the single K2 point clouds, scan position 3 shows the highest coefficient of correlation (0.88) and scan positions 5 and 7 the lowest (0.48).

### Direct derivation of plant heights

When examining the heights of individual plants extracted directly from the point clouds, the results are also indicating a general height underestimation in K2 data and an improvement of plant height derivations by combining K2 point clouds.

**Fig. 6:**
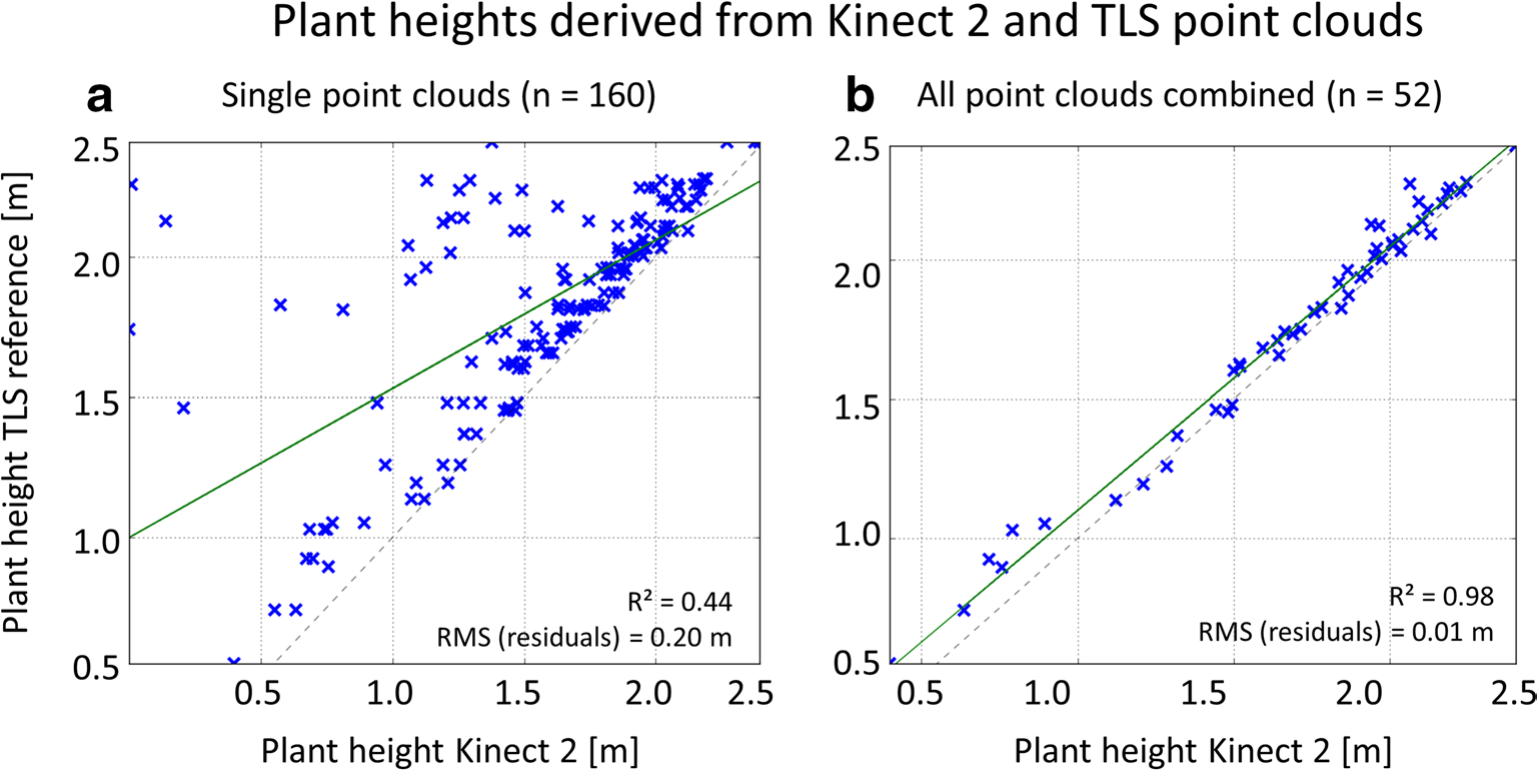
Scatter plots of plant heights derived from K2 and TLS point clouds. **a** Single K2 point clouds. **b** All K2 point clouds combined. *Green line* linear regression line, 1:1 *line drawn dotted in grey*. Note that one plant position can be included in different single frames, leading to multiple extracted plant heights at that position (leading to n = 160 in **a**)

Figure 6 shows the plant heights derived from single K2 point clouds and their TLS reference counterparts according to scenario 2 (plant positions are known). The data pairs extracted from the single point clouds contain pronounced blunders with an underestimation of plant height and an R^2^ of 0.44. Contrary in case of the combined point cloud where plant height value pairs exhibit an R^2^ of 0.98 with K2-based values being slightly lower than the TLS reference.

The plant heights derived via the extraction of local maxima and minima (scenario 1: plant positions are not known) lead to similar results with an R^2^ of 0.96 and an RMS of residuals of 0.01 m in case of the combined K2 point clouds (n = 13), as well as R^2^ = 0.73 and RMS = 0.07 m in case of the single K2 point clouds (n = 44). The number of plant height values extracted for scenario 1 is relatively low because of the restriction that only TLS plant heights within a radius of 0.125 m around a K2 local maximum are taken into account.

## Discussion

### Performance of K2 sensor

The scans of empty scenes in different lighting conditions show that most of the measurement artefacts occur on the FOV edges. Similar to [37], filtering the outermost pixels of the depth image can be recommended to exclude most of the measurement artefacts. Additionally, algorithms to remove remaining measurement artefacts within the FOV volume, such as the statistical outlier filter applied in our study, should be included in studies working with K2 data. The major difference between the data captured in various lighting conditions was found in the number of measurement artefacts. Apart from removing artefacts via filters, a strategy to reduce the number of artefacts can, thus, be to capture data at night which could be achieved by deploying an autonomous mobile system to the fields [38].

Regarding precision and accuracy derived from the lab experiment, the values correspond to the findings of other studies, for example precision values below 0.016 m for distances between 0.5 and 4.0 m [26], or from 0.003 m at 0.8 m distance to 0.016 m at 3.0 m distance [37]. Similar in case of accuracy with, for example, Sarbolandi et al. [26] reporting an RMSE of 0.004 m at a distance of 1.3 m. Subsequently, the used K2 device is considered to capture data of sufficient quality for the derivation of crop height models and plant heights, especially as the results may contain small errors such as residual tilting between K2 and screen despite a thorough measurement setup.

In our workflow, the measurement artefacts along the FOV faces and the distorted FOV corners of far measurements are removed by clipping the K2 data to the extent of the upper FOV defined by the highest plant within the measured scene. We recommend including this pre-processing step in all studies working with K2 data to achieve measurements of high accuracy and precision. Furthermore, the minimum scanning range should be 0.80 m or larger to avoid data gaps in the central FOV area, so that the uppermost parts of the plants are included in the point clouds.

### Direct derivation of crop height models

The results of our field experiments indicate a general underestimation of crop heights similar to Li et al. [15] who directly derive CHMs from ALS data. Also Crommelinck and Höfle [17] report CHM underestimations on the basis of TLS data and CHMs derived from DTMs and DSMs. Contrary, the mean CHM deviation in [39] ranges from an underestimation of 14.55% to an overestimation of 17.95%, with the CHMs derived from TLS point clouds of rice paddies via interpolating DTMs and CSMs. Taking the mean CHM differences (Fig. 5) as an example, the maximum relative CHM underestimation is 11.33% of the crop height when assuming an average crop height of 2.08 m for scan position 8. Subsequently, it has to be decided whether or not an underestimation of that order can be accepted within the frame of a study or application. A possible approach to tackle the deviations can be to apply a removal of systematic errors by empirical, site-specific and crop height-adaptive correction functions that need to be trained with reference samples.

**Fig. 7:**
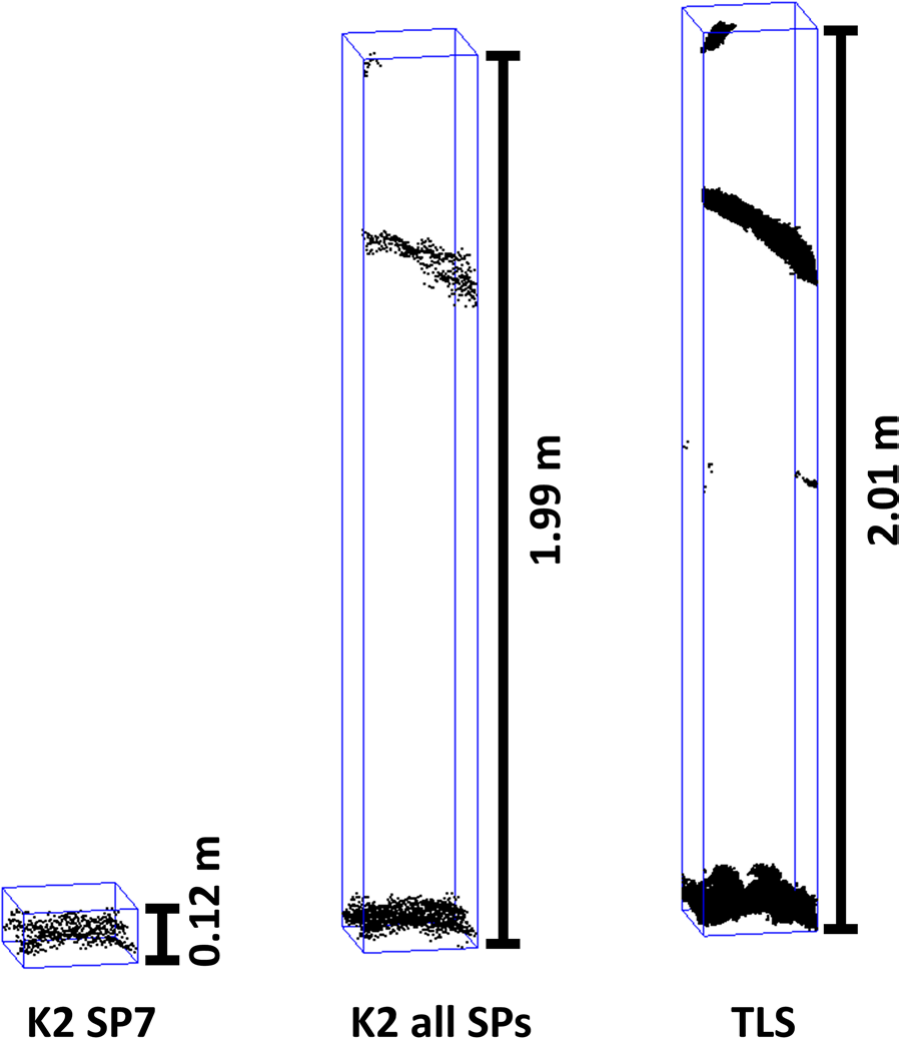
Exemplary point clouds within the same 0.25 m × 0.25 m CHM raster cell leading to a large CHM difference value of −1.89 m. *Left* K2 data captured from scan position 7, *center* data from all K2 scan positions combined, *right* TLS reference point cloud. *Bounding boxes* indicate extent of point clouds

Regarding the influence of pronounced differences in CHM raster cells, we minimize the number of measurement artefacts by applying a statistical outlier removal algorithm. However, some CHM cells derived from K2 data still differ strongly from the TLS reference CHM values, leading, for example, to Q–Q plots including blunders as in [35]. One reason for the occurrence of CHM blunders can be a different coverage of the scanned objects with measurements. Especially in case of the single K2 point clouds, where only one perspective on the scene was captured, effects such as occlusion as well as cutting the data to the upper FOV can affect the derived CHMs (Fig. 7).

Despite single cells containing pronounced differences, the TLS and K2 CHMs generally exhibit high accordance as indicated by high R^2^ values especially for the combined K2 frames. Comparable R^2^ values are reached in other studies such as Tilly et al. [39] (0.72–0.91), or Tilly et al. [40] with same or higher coefficients of correlation (0.88–0.98) for TLS-based crop surface models and manual measurements on an averaged plot level.

**Fig. 8:**
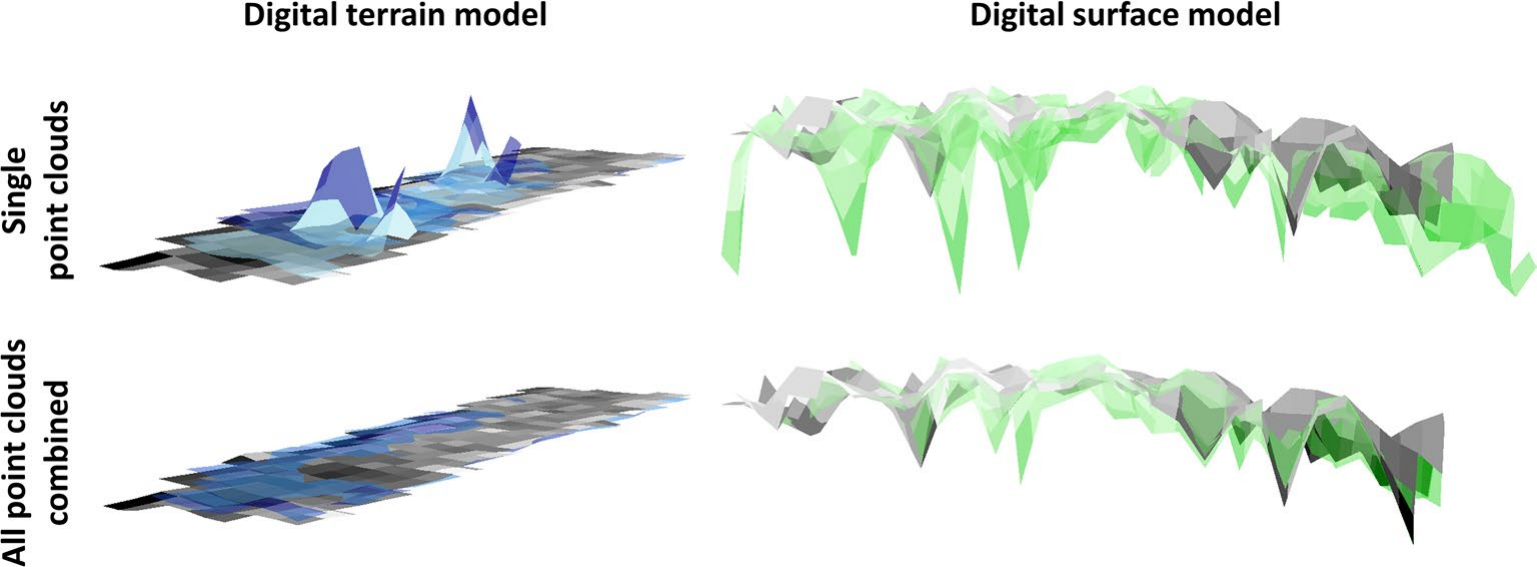
Inclined view on DTMs and DSMs derived from TLS or K2 point clouds. *Grey* TLS reference models, *blue* K2 DTMs, *green* K2 DSMs

Figure 8 exhibits the two compartments DTM and DSM used to derive a CHM. In accordance with Fig. 7, DSM elevation underestimations are attributed to data gaps in terms of upper plant parts. DTM elevation overestimations occur in case the K2 point cloud does not include measurements of the ground, which supports our recommendation to combine multiple perspectives to achieve an advantageous coverage of a crop plot with K2 measurements (Fig. 8).

### Direct derivation of plant heights

**Fig. 9:**
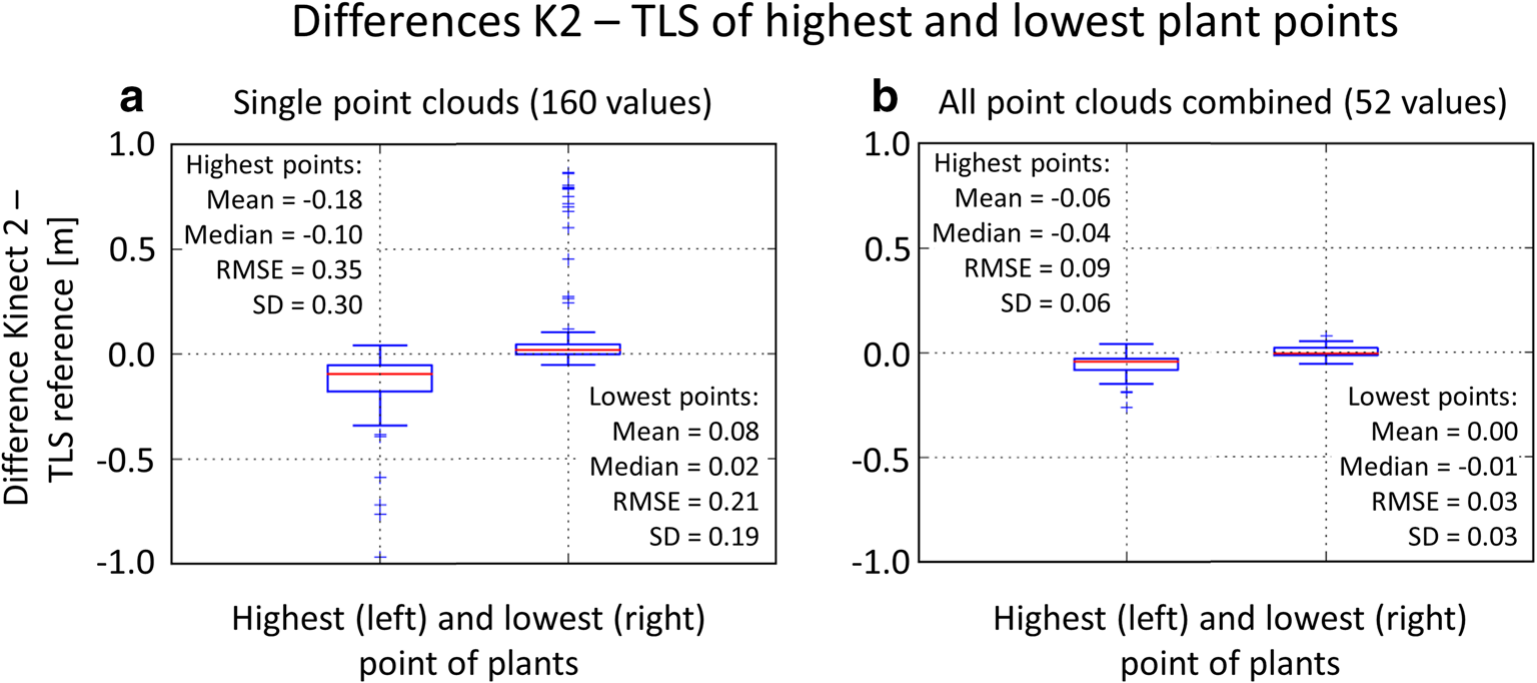
Differences between the highest and lowest points at a plant position extracted from K2 and TLS point clouds. **a** Points extracted from single K2 point clouds. **b** Points extracted from all K2 point clouds combined

Similar, the individual plant heights derived directly from point clouds are calculated via the highest and lowest point at a plant’s position. Figure 9 sums up the differences K2 − TLS between highest points or lowest points, both for the single K2 point clouds and the combination of all point clouds. The differences in the single point clouds lead to pronounced plant height underestimations. Contrary, when the K2 point clouds are combined, the lowest points exhibit very low differences to the TLS reference, whereas the highest points still comprise a range of deviations comparable to the values presented for the median CHM height differences.

**Fig. 10:**
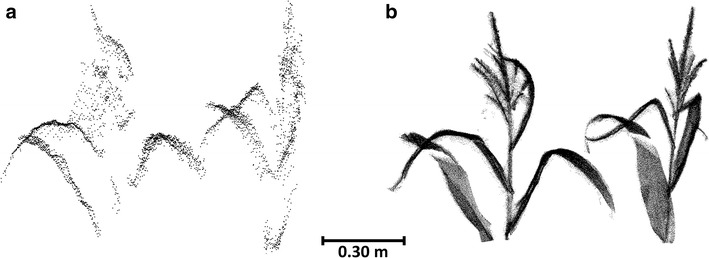
Detailed view on the point clouds of upper part of two maize plants. **a** K2 point cloud captured at scan position 8. **b** TLS point cloud

Generally, the uppermost parts of plants may not be covered in K2 data despite the high resolution of the depth image. Similarly, Grenzdörffer [22] state an insufficient coverage of maize inflorescence in photogrammetric point clouds used as a basis for crop height modelling. On the other hand, the resolution is highest when the sensor is close to the measured object (e.g., 0.0022 m point spacing at 0.80 m range) so that also delicate details at the uppermost plant parts can be captured with the scanning setup applied in this study (Fig. 10). However, as our results show residual differences of the highest points at a plant’s position also after combining all K2 point clouds, the application of plant height correction functions is advisable also for the individual plant approach.

Regarding the methods applied on the field data, the quality of co-registration influences the derived measures: If the tips of plant organs reach into certain CHM cells in case of the TLS reference data but not in case of the K2 data due to a minor relative displacement between the datasets, the respective CHM cells contain different crop height values. Accordingly, also the choice of CHM raster cell size further affects the CHM quality. Similar, TLS and K2 datasets were not captured at the same time so that despite the totally calm weather, movements of the plants may be included in the datasets. In case of the analyses on an individual plant level, the mentioned issue of raster cell size is overcome, but also the determination of plant positions via local maxima as well as the extraction of maxima and minima around a certain position can involve the effects such as the movement of plants.

## Conclusion

In our study we show that deriving crop height models of a maize plot directly from K2 point clouds without the need of prior or supplementary measurements is feasible, offering data of high value for site-specific crop management and precision agriculture. The examined CHMs exhibit a general underestimation of crop height and include some cells with pronounced differences to the TLS reference. Also the derivation of individual plant heights directly from the point clouds comprises plant height underestimations. Combining the K2 point clouds leads to improved plant height estimations also in case of the individual plant height derivation. The combination of point clouds reduces both the underestimation of the maximum plant extent, and the overestimation of terrain elevation. By combining multiple K2 point clouds, differences between K2 and TLS amount to an average underestimation of 0.06 m (3.39% of the mean plant height of 1.77 m) for CHMs and the individual plant heights. To achieve a combination of multiple K2 point clouds in operational use, promising approaches for on-line registration are available [41, 42].

The advantage of fewer measurement artefacts when capturing data in darkness can potentially be exploited by operating autonomous mobile platforms, collecting crop heights at night as a preparation for field treatments the following day. Using unmanned aerial vehicles as platforms can be feasible, but may involve issues regarding downdraft-induced plant movement, especially when the measuring range restricts the platform’s height above ground.

Similar, the maize plot examined in this study required the K2 to be mounted relatively high. Assessing the performance of low-cost devices for different crop types with other growth characteristics in terms of height thus opens further research paths. Also follow-up studies examining crop types with other densities, plant organ morphologies, etc. are of high importance especially regarding the idea of direct CHM derivation, because different crop and plant geometries can have strong influences on the visibility of the bare ground.

In any case, devices such as the K2 can contribute to the analyses of growth dynamics via collecting high-resolution 3D geodata in terms of temporal and spatial resolution [16, 43]. Also further applications, for instance monitoring of soil erosion, can use information derived from data originally captured for CHM monitoring. More general, all processes which include a change in the 3D geometry of natural objects and which can be captured in terms of temporal and spatial scale can profit from low-cost methods producing 3D geodata.

## Additional files

**Additional file 1.** Schematic drawing of K2 FOV and measurement artefacts. **a** Pyramid-shaped FOV edges. K2 sensor corresponds to tip of pyramid. **b** Exemplary measurement artefacts of a frame scanning into an empty volume of air colored according to distance FOV edge–measurement artefact.

**Additional file 2.** Schematic drawing of frontal view on field experiment mountings. **a** K2 mounting with K2 in nadir perspective over maize field, **b** TLS mounting with tilted scanner to account for nadir field of view restriction. (Plant drawing: [44]).

**Additional file 3.** Schematic drawing of the K2 field of view (FOV) and the effect on plants located only partly within the FOV. Plant parts marked in red are not captured, plant parts marked in blue are excluded from the crop height model calculation. (Plant drawing: [44]).

**Additional file 4.** CHM accuracy measures derived from the difference values CHM_K2_ − CHM_TLS_. Rows: K2 scan position, columns: accuracy measure.

## Abbreviations

ALS: airborne laser scanning
CHM: crop height model
DSM: digital surface model
DTM: digital terrain model
FOV: field of view
K2: Microsoft^®^ Kinect^®^ for Xbox One™
NMAD: normalized median absolute difference
Q–Q plot: quantile–quantile plot
RANSAC: random sample consensus
RMS: root mean square
SD: standard deviation
TLS: terrestrial laser scanning
