# Supplementary figures and images for: Direct derivation of maize plant and crop height from low-cost time-of-flight camera measurements
Source: Plant Methods. 2016 Nov 28;12:50. doi: 10.1186/s13007-016-0150-6 (PMC5127001; doi:10.1186/s13007-016-0150-6)

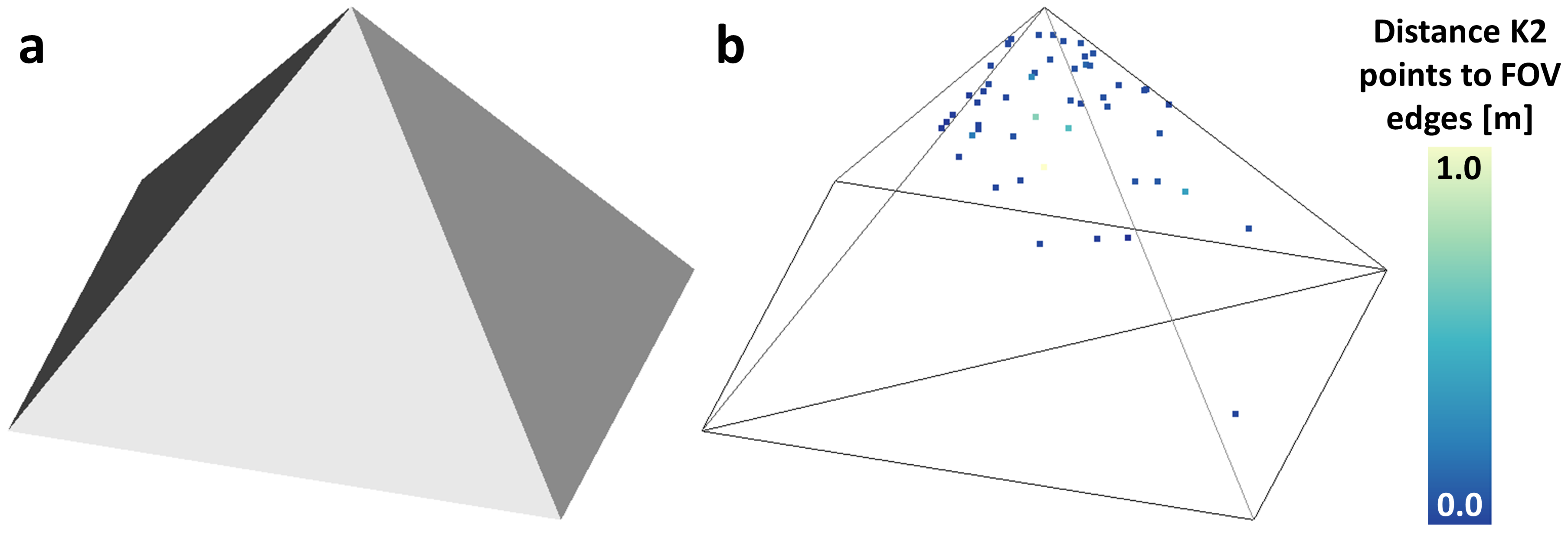

Supplement: Supplementary file 1 — Additional file 1. Schematic drawing of K2 FOV and measurement artefacts. a Pyramid-shaped FOV edges. K2 sensor corresponds to tip of pyramid. b Exemplary measurement artefacts of a frame scanning into an empty volume of air colored according to distance FOV edge–measurement artefact. [file 13007_2016_150_MOESM1_ESM.tif]

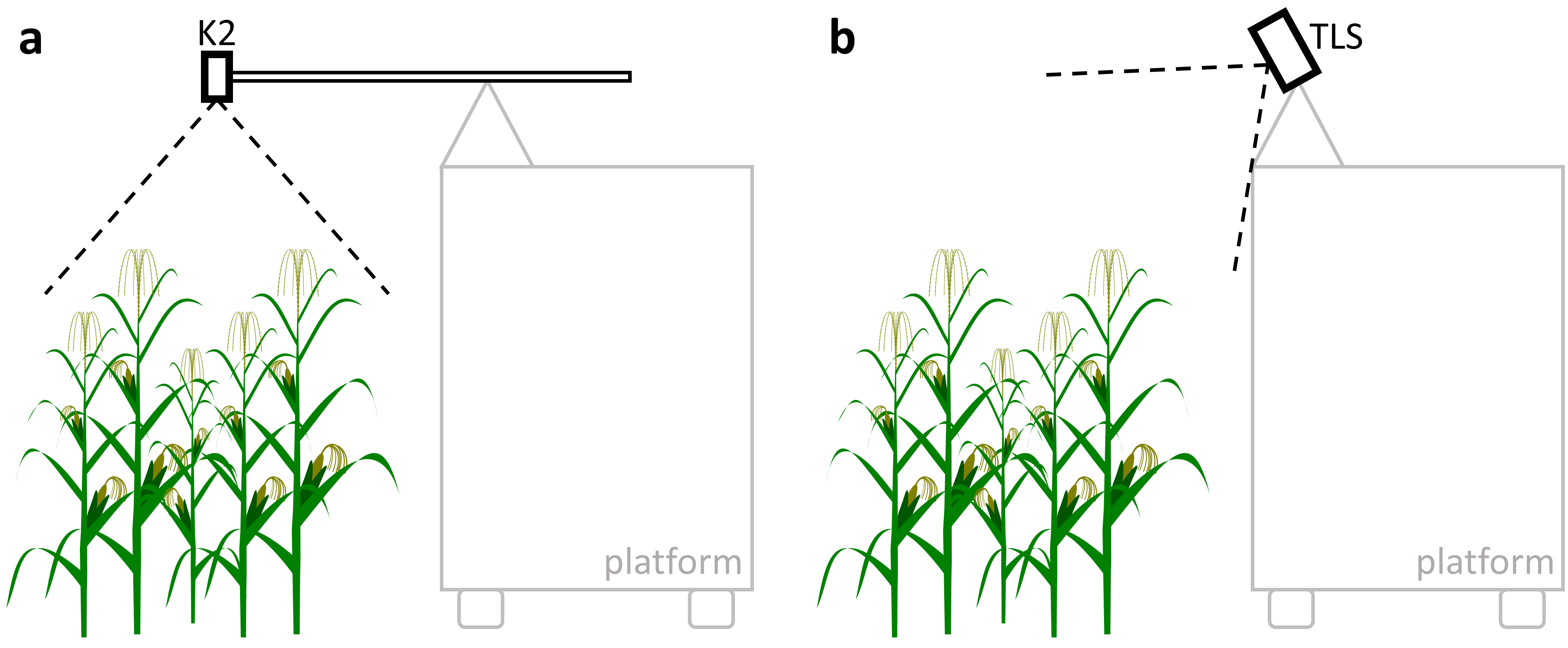

Supplement: Supplementary file 2 — Additional file 2. Schematic drawing of frontal view on field experiment mountings. a K2 mounting with K2 in nadir perspective over maize field, b TLS mounting with tilted scanner to account for nadir field of view restriction. (Plant drawing: [44]). [file 13007_2016_150_MOESM2_ESM.tif]

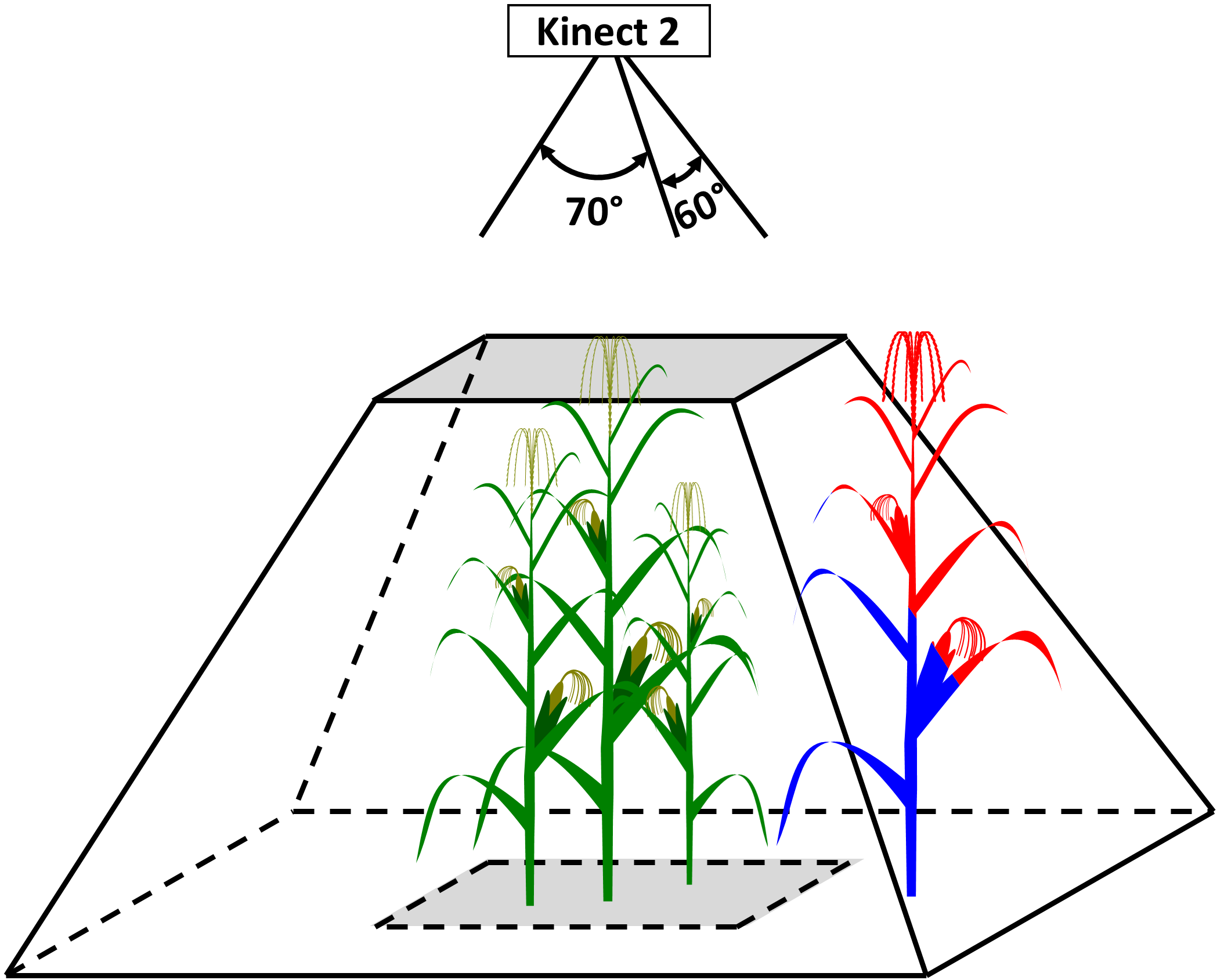

Supplement: Supplementary file 3 — Additional file 3. Schematic drawing of the K2 field of view (FOV) and the effect on plants located only partly within the FOV. Plant parts marked in red are not captured, plant parts marked in blue are excluded from the crop height model calculation. (Plant drawing: [44]). [file 13007_2016_150_MOESM3_ESM.tif]

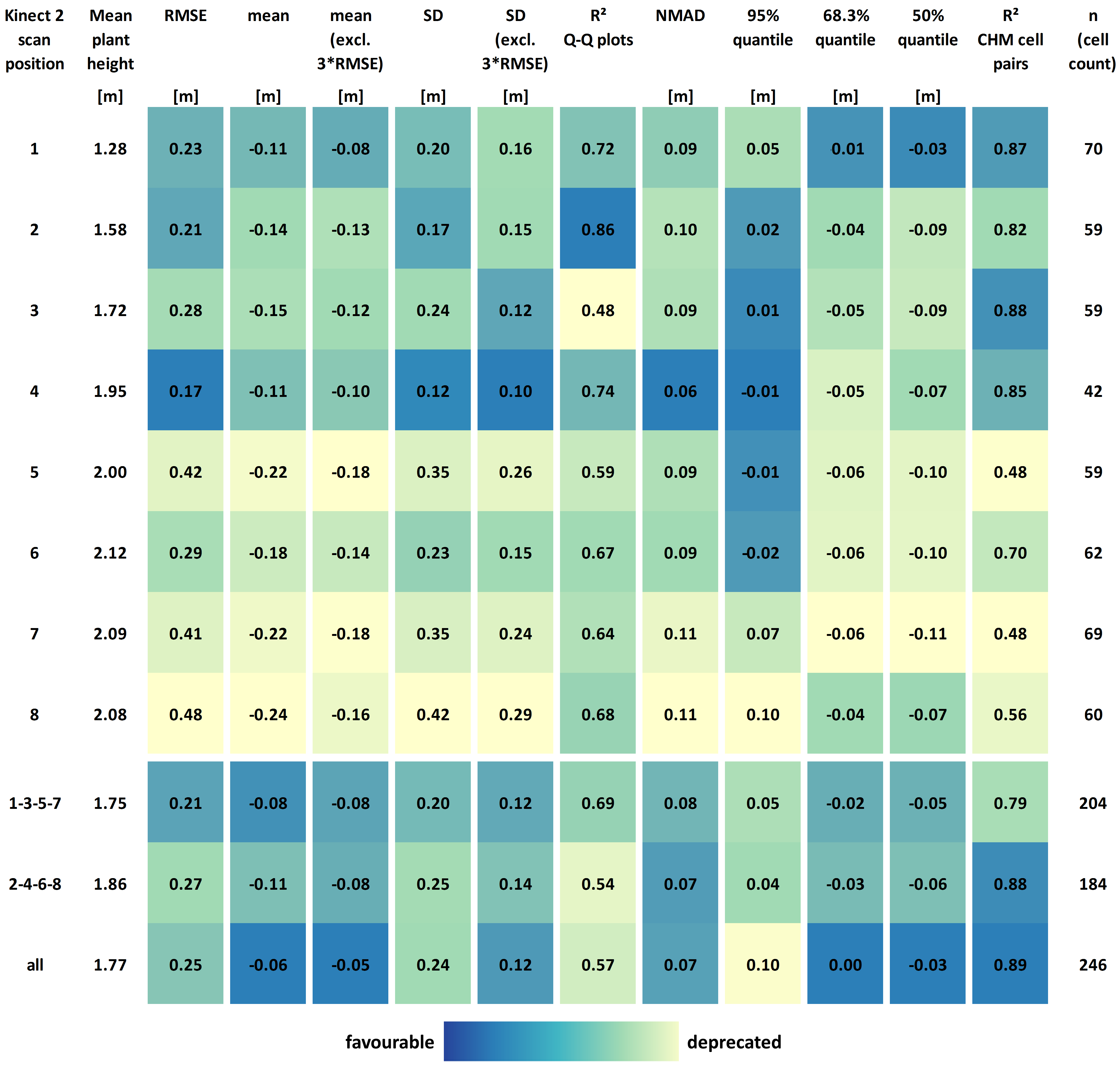

Supplement: Supplementary file 4 — Additional file 4. CHM accuracy measures derived from the difference values CHMK2 − CHMTLS. Rows: K2 scan position, columns: accuracy measure. [file 13007_2016_150_MOESM4_ESM.tif]
